# Assessing the Possibility of Recurrent Diabetic Foot Ulcer Prevention via Remote Patient Monitoring: A Feasibility Study

**DOI:** 10.1002/dmrr.70096

**Published:** 2025-10-17

**Authors:** Caroline A. Abbott, Kerryn J. Franklyn, David E. Stuart, Ellen Kirwan, Sinead Flynn, Ron Scott, Caroline McIntosh, Andrew J. M. Boulton

**Affiliations:** ^1^ Department of Life Sciences Manchester Metropolitan University Manchester UK; ^2^ Diabetes, Endocrinology and Metabolism Centre Manchester Royal Infirmary Manchester UK; ^3^ Rusholme Health Centre Manchester UK; ^4^ University of Galway Galway Ireland; ^5^ Medical City Plano Plano Texas USA; ^6^ Medical City Frisco Frisco Texas USA; ^7^ Department of Medicine University of Manchester Manchester Royal Infirmary Manchester UK

**Keywords:** diabetic foot, diabetic foot ulcer (DFU), digital health, remote patient monitoring

## Abstract

**Aims:**

Our main aims in this study of diabetic patients at risk of foot ulcers were to evaluate: (a) adherence to the use of an at‐home thermal and visual digital foot scanner, (b) the feasibility of utilising thermovisual scan data to perform remote foot assessments, thereby enabling, if indicated, remote intervention by podiatrists and (c) the validity of scanned images to identify skin lesions consistent with those found at a podiatric clinical evaluation.

**Methods:**

In this single arm, open‐label, 12‐week pilot study in two countries, recruited patients with previous diabetic foot ulcer (DFU) (*n* = 27) were asked to stand on a digital foot scanner (OneStep), once a day at home. Plantar thermal and visual scan data were transmitted daily to a centralised monitoring service for daily review. Any abnormalities were immediately reported to the patient's podiatric healthcare provider, who determined appropriate intervention. Primary endpoints were patient adherence, device utility and data validity.

**Results:**

All participants with an active device (*n* = 26) took thermal and visual scans on 1547 days during 1940 active study days, averaging 5.3 ± 1.4 scans/week, with 80 ± 19% adherence (days scan recorded/days in study*100). Visual scans correctly identified all incident DFUs (*n* = 7). Podiatrists agreed that scans enabled the identification of skin integrity issues earlier than standard care (in 82% cases), finding visual scan images useful in 90% of reports and thermal data in 12%. Remote visual assessments agreed well with gold‐standard podiatric examinations in identifying skin integrity risks (kappa = 0.67 [95% CI, 0.53–0.82, *p* < 0.001]), also showing good sensitivity (80%) and specificity (100%).

**Conclusions:**

Remote foot scanning was easy to perform and was used consistently by vulnerable patients. Scans were useful for remote podiatric foot assessments and interventions, and visual images identified DFUs/skin problems to a good level. We now aim to test this monitoring system in a larger scale randomised controlled trial for DFU prevention.

## 1 Introduction

Despite recent advances in medical therapies, the prevalence of diabetes mellitus and diabetes‐related complications continues to increase. With an estimated annual average incidence of > 2% and a life‐time prevalence of 19%–34%, foot ulceration is one of the most common complications in people with diabetes [1, 2]. Diabetic foot ulcers (DFUs) frequently become infected, causing significant morbidity and hospitalisation, and can lead to lower‐extremity amputation, creating a negative impact on health‐related quality of life and mobility [3, 4, 5, 6]. The burden on health care systems is large with the average cost of treating a DFU over £8000 in England, and the NHS spending over £1.2 billion each year on the problem [7]. Furthermore, mortality risk doubles at 10 years in patients with a DFU history compared with those without [8].

Recognising the above, experts and international guidelines have recommended the widespread establishment of preventative foot care programmes for people with diabetes, calling for foot screening, education, frequent podiatry visits for at‐risk patients, protective pressure relieving footwear, rapid response when an ulcer is detected, and the establishment of multi‐disciplinary foot care teams [9, 10, 11, 12, 13, 14]. Current guidelines also advise encouragement of patients to perform daily visual self‐examinations to empower them to assist in the care of their own feet [13, 15]. Groups such as the International Working Group on the Diabetic Foot (IWGDF) have also recommended temperature self‐monitoring for the prevention of recurrent foot ulcers [13, 14]. However, to date, the technique has not been widely adopted potentially due to the burden placed on patients for manual self‐testing and the complexities of logging their own data ([16]). Despite the preventative foot care guidelines, DFUs have a high re‐ulceration rate, with up to 40% of patients developing a subsequent DFU in the first 12 months following healing [2].

Remote digital health technologies designed to screen for biomarkers of DFU and assist people with diabetes in preventive self‐care and evaluation of their feet have undergone rapid growth in development in recent years, with varying degrees of validation and success [17, 18, 19]. Indeed, since the COVID‐19 pandemic, new proposed models of care have emphasised the development of remote consultations, home monitoring and more, using such digital technologies, with the aim of reducing the number of ‘in‐person’ consultations for highest‐risk patients [20, 21, 22, 23, 24].

In this study, we investigated the feasibility of using a remote home‐use foot scanning system (Bluedrop Monitoring System [BMS]), comprising a plantar foot temperature monitoring facility and the ability to take plantar foot photographic images, designed to be easy to use and integrate well with the healthcare system. To our knowledge, this is the first remote monitoring device system which combines the ability to assess both visual and temperature data. Given the novelty of the BMS, a feasibility study was a necessary first step. No prior system has evaluated this dual‐modality approach in high‐risk patients in a home setting. Before testing efficacy or cost‐effectiveness in a larger trial, it was important to establish whether the system is usable, produces clinically useful data, and integrates well into routine care. This aligns with best practice for phased intervention development.

Our primary objective was to track and evaluate patient adherence to the daily usage of the at‐home digital foot scanning system. We hypothesised that the at‐home foot scanner would be easy to use and used consistently by the patient. Our secondary objectives were: to assess the feasibility of utilising the thermovisual scan data to perform a remote assessment and enabling remote intervention by the podiatric healthcare provider; to quantitatively assess the ability of visual scan images to remotely identify skin issues consistent with that found at a podiatric clinical evaluation, that is to show validity and robustness of the visual images; to track the number of and severity of DFUs that develop over the course of the study; and to examine the feasibility of the approach intended to be used in a larger scale randomised controlled trial for DFU prevention using this monitoring system alongside standard podiatric practice.

## 2 Materials and Methods

### 2.1 Study Design and Participants

This single‐arm, open‐label pilot study was conducted in two countries (Ireland and UK), with patients recruited from one multidisciplinary outpatient diabetic foot clinic from each country. Major inclusion criteria were: type 1 or type 2 diabetes, aged 18 years or older, history of DFU present for at least 2 weeks and healed for at least 2 months before study entry, vibration perception threshold (VPT) ≥ 25 V at either foot, ability to walk independently for 10 m or more, receiving regular footcare from a podiatrist and access to a mobile phone so able to receive text notifications. Major exclusion criteria were: active foot ulceration, weight > 150 kg, active Charcot neuro‐osteoarthropathy, active foot infection, history of major or minor lower limb amputation excluding up to 3 toes (hallux toes intact), significant clinical evidence of peripheral arterial disease including history, symptoms and signs (absent pedal pulses) and severe physical or mental condition(s) that limited the ability to follow instructions for the study based upon clinical judgement. All patients provided written informed consent before recruitment and received routine standard podiatric care throughout the study comprising regular clinical visits, education, and preventative foot care/podiatry, as required.

The study was prospectively registered on clinicaltrials.gov (NCT05039645). All relevant ethical and regulatory approvals were obtained in the UK and Ireland for this clinical investigation prior to study start (Irish REC refs: C.A.2614 20th Apr 2021/22‐NREC‐MD‐007 16th Mar 2022 and UK REC ref: 21/NW/0190 4th Mar 2022). All activities were carried out in accordance with the Declaration of Helsinki and applicable guidelines for Good Clinical Practice (GCP). All participant records underwent Source Data Verification (SDV) and monitoring.

### 2.2 Protocol Overview

Recruited participants were provided with a OneStep Foot Scanner device (in the form of bathroom weight scales) and a portable cellular internet router, for use at home. Patients were instructed to take a daily foot scan for up to 12 weeks. After each scan, data were transmitted automatically from the foot scanner to a cloud‐based server, for review by an independent remote study Monitoring Service. If skin thermal/visual abnormalities were identified on the scans, the scans were flagged and reports were sent, via the Central Study Coordinator, to the study site for podiatric review and appropriate footcare intervention. Patients were withdrawn from the study if they developed a DFU (ranging from superficial to full‐thickness ulcers) according to (Texas classification) [25].

Bluedrop Medical provided study devices and funding to support the conduct of this clinical investigation and the data analyses.

At the participant's baseline visit, the study podiatrist assessed peripheral neuropathy status using Vibration Perception Threshold (VPT) testing and the modified neuropathy disability score (NDS) [1]. Vascular status was determined by palpation of the pedal pulses of both feet and checking for a history of revascularisation. Feet were visually inspected for signs of skin damage, emerging or active DFU, infection or deformity. Medical records were reviewed to collect variables relating to previous medical history and DFUs. Following recruitment, instructions were given to the patient on how to set up and use the OneStep device and portable cellular internet router once it had been delivered to their home address.

At the end of the study, all patients attended a final visit.

The investigational device system (BMS) comprised the home‐use OneStep Foot Scanner (also known as Delta Foot Scanner) device and accompanying Sentinel Review Interface (SRI) software (Bluedrop Medical Ltd., Galway, Ireland), enabling the Remote Monitoring Service to assess participants' feet, daily, for thermal and visual signs of skin inflammation or damage.

**FIGURE 1:**
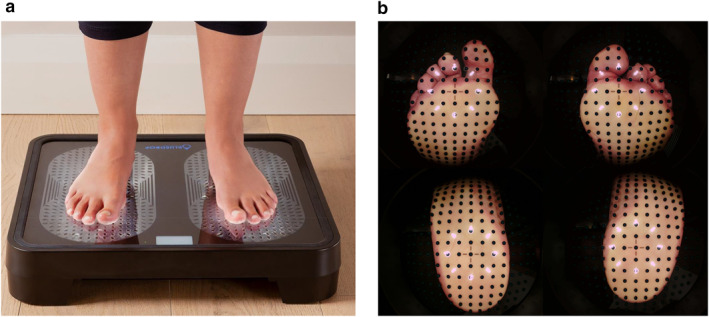
(a) OneStep device, designed to look and behave like a standard home weight scale, taking the Patient 30 s to use per day at home. (b) Visual monitoring scan image from SRI software. The black dots are the patented eTLC (thermochromic liquid crystal) temperature sensors providing the thermal readings.

The OneStep device took the form of bathroom weight scales (Figure 1a) containing a high‐density array of over 700 discreet temperature and photo image sensors plus LEDs embedded within the surface panel. Participants were instructed to set up their scanner on a hard floor at home, then advised to step barefoot onto the device just once per day, whenever convenient, for 30 s, during which time an automated thermovisual scan took place. Patients were not advised to limit weight‐bearing activities for a specific period prior to each scan as this may have adversely affected adherence to device use. The resulting scan contained 4 High‐Definition visual images of the plantar surface of the feet as well as temperature data recorded from the 700 temperature sensors (± 0.6°C accuracy). Scan data were automatically transmitted wirelessly for next‐day analysis, including on both weekend days, by expert, trained clinical observers (a medical doctor and a registered nurse) in the remote Monitoring Service. An example of scan visual images is shown in Figure 1b.

The SRI software included a web application which provided the remote clinical observer with access to the specific, anonymous, thermovisual patient transmitted data. All communication/data from the device to the cloud services were encrypted at rest and in‐transit according to cyber security best practice. Data and services access were locked down to authenticated/authorised users. Patient identifiable information was tagged, encrypted and scans were anonymised.

Each study day, the Monitoring Service observer analysed participants' scans using the web application. Six pre‐defined points on each foot were selected for temperature assessment, most often at the hallux, 1st, 3rd, and 5th metatarsal heads, midfoot and heel [26, 27], which is an evidence‐based clinically validated method to balance specificity and false‐positive rate. The points were selected manually by the observer; the software then compared the points on one foot to the same points on the opposite foot. If paired points had a temperature difference (delta) exceeding the defined delta threshold (≥ 2.2°C) on two consecutive daily scans, this was defined as a ‘hotspot, ’ the scan was flagged, and a report was generated for review by the local site clinical team. This consecutive day approach to delta change mirrors protocols from previous studies [16, 28], designed to minimise false positives while maintaining sensitivity to pre‐ulcerative inflammation. Similarly, a report was also generated for review if there were any visual signs of skin damage on the scan. Reports were also created for patient education purposes, addressing any foot hygiene issues and foot misplacements on the scanner. Reports contained photographic images of the feet, thermal information from selected points, plus observer notes regarding the flagged scan, and were emailed to the Central Study Coordinator within 24 h. Reports were then personalised and emailed to the study site podiatrist within 24 h.

Local site podiatry reviewed the images and report provided, contacted the patient by telephone by the end of the next working day, and then implemented an appropriate course of action, which would include ongoing remote monitoring, advice upon activity reduction, physical checks of foot/feet, weight off‐loading, or, if considered more urgent, an in‐clinic appointment for foot assessment.

All patients received automated text notifications via the SRI software. Good adherence to daily scanning was acknowledged, and reminders to scan were triggered if no scans had been received for 3 days. The Central Study Coordinator contacted each patient via a monthly phone call to perform check ins, administer surveys and as a point of contact for any troubleshooting.

The primary study endpoint was patient adherence, that is, the percentage total number of days on which at least one scan was recorded/the total number of days the participant was actively in the study. This was calculated for all participants with access to an active foot scanner at home, including those lost to follow‐up. Mean adherence for all patients was calculated to determine overall adherence across the study.

Adherence categories were assigned as: ‘High adherence’: used the device ≥ 3 times/week; ‘Medium adherence’: used the device between ≥ 1 and < 3 times/week; ‘Non‐adherence’: used the device < 1 times/week. The number and percentage of patients who had high adherence/medium adherence/non‐adherence at each week were calculated.

**FIGURE 2:**
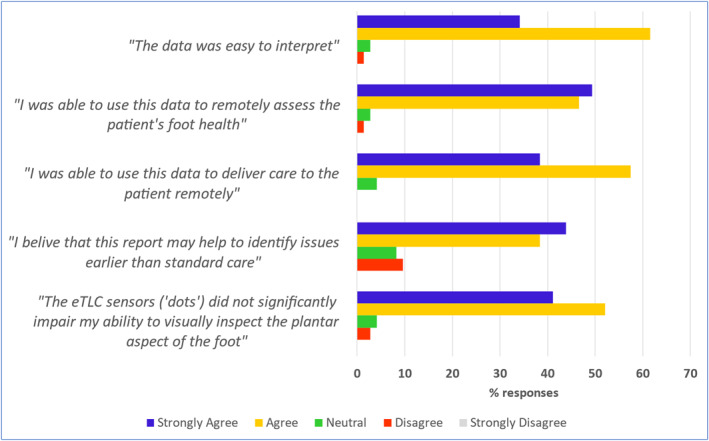
Report utility statements, completed by study podiatrists. The clinical usefulness of the thermovisual data from reports (*n* = 73) to remotely assess and issue guidance to the patient was assessed using a 5‐point Likert scale to grade podiatrists' responses to statements. Data is presented as the percentage number of reports in each Likert response category.

**FIGURE 3:**
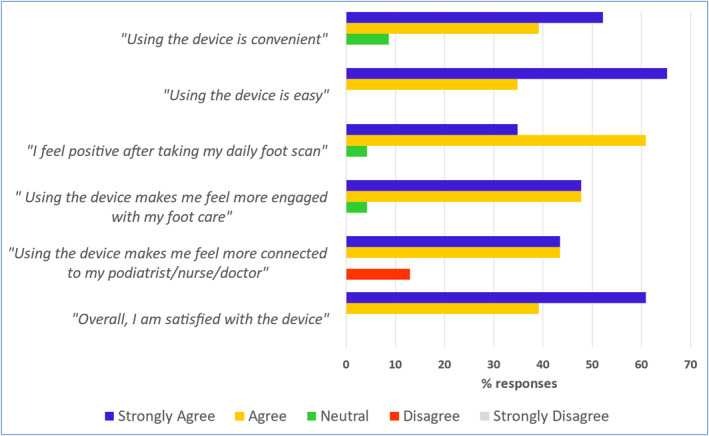
Device usability statements, completed by the study participants. The usability of the device was assessed in study participants (*n* = 23) using a 5‐point Likert scale to grade participant's responses to statements. Data are presented as the percentage number of reports in each Likert response category.

The secondary endpoint measures were as follows:

- Utility of the thermovisual data. The local site podiatrist completed a brief survey to assess the overall usefulness of each report for (a) remote analysis and (b) remote intervention. For each statement there were 5 response options on a Likert scale: Strongly Disagree, Disagree, Neutral, Agree and Strongly Agree (Figure 2).
- Device usability. At the end of the study, participants were asked to what extent they agreed with statements about the device, covering themes of convenience, ease of use, attitudes towards care and overall satisfaction. For each statement there were 5 response options on a Likert scale: Strongly Disagree, Disagree, Neutral, Agree and Strongly Agree (Figure 3).
- Data validity for assessing skin integrity. At the end of the study, the level of agreement between the gold‐standard, clinical podiatric examination of the foot and the remote monitoring service examination of an equivalent scan, was investigated by analysing data collected from a sub‐cohort of participant visits at the UK site (*n* = 11). Visit data were included in validity analyses if the participant's clinic visit and home scan image were both taken within a maximum 4 days of each other. Twelve specific plantar areas per foot had been assessed by the podiatrist during each study visit, and skin integrity was recorded as: (1) DFU, (2) ‘area of concern’ (subcutaneous bleed, extravasation, pre‐ulcerative lesion, blister, etc.), (3) callus (mild, moderate or severe) or (4) no concerns. Similarly, an equivalent, remote foot scan (± 4 days from each podiatry foot assessment) was assessed and categorised for skin integrity, as above, by a trained, remote observer. The observer assessed each scan image on a computer screen, blinded, during an online meeting in the presence of the research coordinator who recorded the observer's categorised assessment at each plantar area. If observers noted a bandage/dressing at a particular site, this was also considered an ‘area of concern, ’ in addition to the above descriptions. This method was repeated for a second observer.

### 2.3 Statistical Analysis

Descriptive statistics of continuous variables were reported as mean (SD) or median (IQR). For categorical variables, proportions and frequencies were given.

The sample size of *n* = 30 was based on the following assumption: sample sizes between 24 and 50 have been recommended for feasibility studies of patient adherence such as this [29]. With a sample size of 30, we were able to estimate an adherence rate of 80% to within a 95% confidence interval of ± 14.3%. The width of the confidence interval (in %) was calculated using the formula: 1.96 × √(*p* × (1 − *p*)/*n*), where *p* was the percentage we expected to see (80%), and *n* was the intended sample size (*n* = 30). This would enable us to make decisions in planning future larger clinical trials based upon the ‘true’ adherence rate (± ∼15%).

The level of agreement between the gold‐standard podiatric foot examination and the test remote scan assessment, for identifying significant skin integrity problems at 264 sites, was determined using Cohen's kappa. An ’area with DFU’ and an ‘area of concern’ were combined for analysis as both are clinically significant events, which would trigger generation of a report. Statistical evaluation of the sensitivity and specificity of the test method against the gold‐standard method was performed for both observers.

A *p* value of ≤ 0.05 was considered statistically significant. All analyses were done using IBM SPSS, version 28.0.1.1.

## 3 Results

The study was conducted from March 2022 to December 2023. Thirty‐one participants gave consent, with 27 participants enrolling as 4 became ineligible before study start. Sixteen participants completed the 12‐week period (89.3 ± 8.9 days [mean ± SD]) and 11 participants ended the study earlier (59.2 ± 22.3 days) due to incident DFU/foot skin lesion (*n* = 7), device technical issues (*n* = 2), becoming lost to follow‐up (*n* = 1) or failing to set‐up the device after 6 weeks (*n* = 1),

The baseline characteristics of the 27 participants are listed in Table 1. Patients had a relatively high age and duration of diabetes, with poor glucose control, a high prevalence of hypertension, retinopathy and other co‐morbidities, and a high level of foot deformities, with two‐thirds having a smoking history. Anti‐hypertensives and statins were prescribed to 78% of this cohort and 33% regularly took medication for neuropathic pain. All participants had moderate to severe peripheral neuropathy, with both large and small fibre dysfunction.

**TABLE 1:** Baseline characteristics of the study participants.

Characteristics	
*n*, Galway site: Manchester site	17 (63%): 10 (37%)
Age (years)	66.0 ± 10.4
Male	22 (81.5%)
Type 2 diabetes	18 (66.7%)
Diabetes duration (years)^a^	21.4 ± 14.7
Ethnicity	
White British	11 (40.7%)
Asian British	1 (3.7%)
White Irish	15 (55.6%)
HbA1c %^b^	7.5 (6.5–8.6)
(mmol/mol)	(57.5 (47.0–68.5))
BMI (kg/m^2^)	29.1 ± 4.2
Co‐morbidities	
Nephropathy	12 (44.4%)
Retinopathy	9 (33.3%)
Hypertension	17 (63.0%)
IHD	5 (18.5%)
Other	
Smoking status	
Current	4 (14.8%)
Ex	14 (51.9%)
Never	9 (33.3%)
Take alcohol	14 (51.9%)
Corrected vision	18 (66.7%)
Medications	
Insulin	14 (51.9%)
Glutides	5 (18.5%)
Metformin	12 (44.4%)
Antihypertensives	21 (77.8%)
Statins	21 (77.8%)
IHD drugs	16 (59.3%)
Neuropathic pain drugs	8 (29.6%)
Neuropathy measures	
NDS^c^	8.3 ± 2.2
VPT (V) (mean of both feet)^b^	39.9 ± 6.5
Foot deformities (R or L)	
Claw toes	12 (44.4%)
Prominent metatarsal heads	11 (40.7%)
Bony prominences	11 (40.7%)
Plantar callus	17 (63.0%)
Charcot	3 (11.1%)

*Note:* Data are *n* (%), mean ± SD or median (IQR).

Abbreviations: IHD, Ischaemic heart disease; NDS, Neuropathy Disability Score; VPT, Vibration Perception Threshold.

^a^ 26 patients with data.

^b^ 24 patients with data.

^c^ 23 patients with data.

There was one adverse event recorded during the study that was determined to be unrelated to participation in the clinical investigation.

Participants took at least one daily scan on 1547 days, during 1940 active study days. There were 98 days during the study when participants advised in advance that scanning would not be possible because of holidays, or when technical support was required, and these were removed from the active study period, to accurately assess patient adherence to using the device when it was readily available to them.

Adherence to daily device use was calculated from data from 26/27 participants. Adherence calculations included all participants except one who failed to ever set up their device at home from study onset and therefore had no access to the active device. Overall participant adherence was 80 ± 19% (mean ± SD), that is, patients used their device on 80% of all their active days in the study. On average, participants used the device 5.3 ± 1.4 times per week during the entire study period.

**FIGURE 4:**
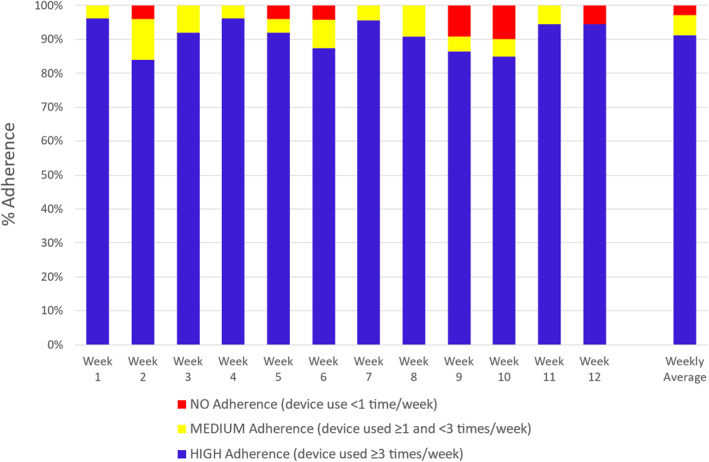
Percentage number of patients with high, medium or no adherence to using their foot scanner device for each study week. Participant data: Week 1, *n* = 26; Week 2, *n* = 25; Week 3, *n* = 25; Week 4, *n* = 26; Week 5, *n* = 25; Week 6, *n* = 24; Week 7, *n* = 23; Week 8, *n* = 22; Week 9, *n* = 22; Week 10, *n* = 20; Week 11, *n* = 18; Week 12, *n* = 18.

Twenty‐four (88.9%) participants had average high adherence at each week, 2 (7.4%) had medium adherence and 1 (3.7%) had no adherence (failed to set up the device). Furthermore, the mean percentage number of participants with weekly high adherence across the whole study was 91.2% (Figure 4).

The Monitoring Service generated 73 reports (from 25 participants) after detecting the following scan abnormalities (*n* = 77): a new area of concern or potential DFU, *n* = 19 (24.7%); presence of a new bandage, *n* = 17 (22.1%); callous build up, *n* = 15 (19.5%); foreign body/material to be removed, *n* = 9 (11.7%); thermal ‘hotspot’, *n* = 6 (7.8%); poor foot hygiene, *n* = 6 (7.8%); incorrect foot placement/wearing socks, *n* = 3 (3.9%); reddened area, *n* = 1 (1.3%); escalation after no response *n* = 1 (1.3%).

Of the 73 reports, 3 were created exclusively due to the presence of excessive calluses or ‘foreign bodies’ and therefore temperature assessments were not performed. Of the remaining 70 reports, 778 temperature measurements were made from a potential maximum of 840 (12 measurements per two feet), giving a temperature data capture of 92.6%. There was slight loss in data capture due to some instances of heels or great toes not making full contact with the scanner due to foot deformities, and bandages and/or trousers obscuring areas of the plantar surfaces.

Local site podiatry teams took 1.1 ± 1.9 days (mean ± SD) to respond after receiving a generated report. Podiatry intervention decisions were as follows: take a ‘wait and see’ approach and continue to monitor future daily scans remotely (37.0%); offer remote patient advice over the telephone on how to treat the issue (24.7%); instruct patient to come to clinic for a non‐emergency appointment (28.8%); instruct patient to come to clinic for an emergency appointment (urgent cases) (9.6%).

Seven incident DFUs occurred throughout the study, in 7 participants (25.9%), at plantar (*n* = 3), dorsal (*n* = 2), lateral dorsal (*n* = 1) and interdigital (*n* = 1) sites. All these 7 DFUs were either identified (*n* = 6) or pre‐empted shortly prior to ulceration (*n* = 1) by the monitoring system following inspection of patients' visual scan images. For these 7 cases, flagged reports were generated due to the appearance of bandages (*n* = 5), severe callus (*n* = 1) and an apparent open wound (*n* = 1), resulting in clinic visits (4 emergency and 3 non‐emergency) being made. At these clinic visits, the presence of DFUs was confirmed in 6 cases and a pre‐ulcerative lesion was identified in 1 case which, despite intensive intervention by the podiatry team, developed into a DFU 2 weeks later. Foot temperature changes were not noted on any of the scans for these cases. This was also the first point of detection for 4 of the new DFUs. Causes of these 4 previously undetected DFUs were: (a) self‐inflicted injury with a scalpel whilst removing callus (1st MTH, grade 1A); (b) blunt trauma whilst swimming (hallux, grade 1A); (c) abrasion from a slip‐on shoe (dorsal toe, grade 1A) and (d) cause unknown (4th/5th interdigital space, grade 1B).

Podiatrists strongly agreed/agreed that they could use report scan data to remotely assess patients' foot health in 70/73 (96%) cases (Figure 2); furthermore, podiatrists strongly agreed/agreed in 60/73 (82%) cases that scans had helped to identify issues earlier than standard care.

Podiatrists found visual scan data useful for their clinical assessments in 66 (90%) reports, examining historic scans useful in 20 (27.4%) reports, temperature scan data useful in only 9 (12%) reports, and patient compliance data useful in only 4 reports (5.5%).

Ninety‐six percent of study participants who completed a device usability statement at the end of study (*n* = 23) strongly agreed/agreed that using the device made them feel, (a) more engaged with their foot care, and (b) confident that their feet were receiving the best possible care (Figure 3). Only 13% disagreed that using the device made them feel more connected to their podiatrist/nurse/physician. All study participants agreed that they were satisfied with the device and found it easy to use.

Within the validity sub‐cohort, in‐clinic podiatric examinations identified 2 plantar areas with DFU, 2 areas with extravasation/1 site with eschar (i.e., 3 ‘areas of concern’) and 17 areas of significant callus. There was a ‘good’ level of agreement between in‐clinic podiatric examinations and Observer 1 remote visual scan assessments, for categorising patients' plantar skin integrity (kappa = 0.67 [95% CI, 0.53 to 0.82, *p* < 0.001]) and a similarly ‘good’ level of agreement between podiatry assessments and Observer 2 remote visual scan assessments (kappa = 0.63 [95% CI, 0.48 to 0.78, *p* < 0.001]). Inter‐observer agreement between remote visual scan assessments (Observer 1 vs. Observer 2) was ‘good’ (kappa = 0.68 [95% CI, 0.55 to 0.82, *p* < 0.001]). The sensitivity of remote visual scan image analysis to identify a real DFU/area of concern requiring intervention, as determined by the in‐clinic study podiatrist, was high, at 80%, for both Observer 1 and Observer 2, respectively. Furthermore, the specificity of the remote visual scan (ability to correctly identify areas without concern) was 100% for Observer 1% and 99.2% for Observer 2.

## 4 Discussion

We have demonstrated the feasibility of a novel, remote visual and thermal foot scanning and monitoring system for potential use in the healthcare system for care of patients at high risk of recurrent diabetic foot ulceration, alongside their standard podiatric care. In brief, participants showed a high level of adherence to device use at home. Podiatry teams reported high levels of satisfaction with thermovisual scan data to remotely assess and issue guidance to the patient and patients reported high levels of satisfaction with device usage. Importantly, the system was validated in its ability to identify skin issues effectively when compared with the gold‐standard in‐clinic podiatry assessment. Visual scan images correctly identified all incident DFU and were the first point of detection for over half of these new DFU cases, ahead of standard podiatry service knowledge. Temperature data was less useful than visual images for enabling remote podiatric foot assessments and subsequent interventions.

Patient adherence to using the BMS device was high, and similar to that found in a remote foot‐temperature monitoring system feasibility study elsewhere in which a high‐risk patient cohort used their smart mat 5 times/week, with 86% participants (*n* = 26) using the system at least 3 days/week over 34 weeks [17]. Indeed, in our study, patients used the foot scanner over 5 times/week, with 91% using the device at least 3 days/week over the whole study. As a high level of patient engagement and adherence is beneficial for intervention studies for plantar DFU healing and recurrence prevention [17, 27, 30], the BMS monitoring system may be considered a suitable intervention for DFU if confirmed in a larger RCT, potentially enabling its availability to a broad spectrum of community‐based diabetic patients at risk of DFU.

There are several potential explanations for the good adherence to device use here. Firstly, this may reflect the quick and simple automated scanning process of the BMS, ultimately causing minimum burden for the patient. Adherence is critical in this era of increasing reliance on AI in healthcare, with cohorts similar to ours having potential struggles with engagement with digital technology, compounded by their many co‐morbidities, plus manual dexterity and eyesight issues [19]. In a multi‐centre RCT intervention study using infrared thermography (DIATEMP study), there was a lower‐than‐expected adherence to remote self‐assessment [16], explained as caused by the daily burden for patients to perform their own foot temperature assessments and log their own results. This ‘prevention paradox’, whereby patients reduce their adherence to self‐assessment once they record their own negative foot issue [22], did not occur here. Secondly, it is likely that our participants became more empowered to self‐manage their footcare through effective use of the digital technology, i.e. easily standing on the device each day; indeed, empowerment and self‐managing health are requirements for preventing DFU [31]. Thirdly, there were efficient communication methods (phone and text messages) between participants and the research coordinator and short response timelines for device trouble‐shooting issues, which minimised potential time out of study. Fourth, regular, human‐touch phone calls were likely to have positively empowered participants. We did not anticipate some of scan image problems that occurred occasionally during study, including feet obscured fully or partially by socks being worn, presence of hanging trouser hems, other foreign objects, large amounts of dirt and debris and poor foot placements on the scanner; however, these ‘real‐life’ occurrences were almost always rectified by follow‐up educational phone calls to the patient.

Nearly all participants reported that they felt confident that their feet were receiving the best possible care, they were more engaged with their own footcare, the device was easy to use, and they were very satisfied with the device. Some expressed anecdotally that they would have enjoyed positive daily feedback from their foot care team after each scan with no issues. Remote daily monitoring systems could therefore include provision of daily feedback to satisfy patients who require regular foot‐health reassurances.

Podiatrists rated the value of the thermovisual scan data highly for making clinical intervention decisions. Podiatrists were able to provide remote patient advice (specifically, advice regarding appropriate footwear and off‐loading, new dressings which had appeared, instructions to make contact with their local podiatry service, poor foot hygiene, presence of foreign bodies such as stones or coins) or ‘wait and see’ and continue to remotely monitor the patients (regarding, e.g., presence of potential foreign bodies, unusual skin colour changes) for > 60% of flagged issues. This indicates the potential of the system to reduce the frequency of ‘in‐person’ consultations for the highest‐risk patients and fulfilling requirements for new models of diabetes foot care [20, 21]. Although ‘wait and see’ remote monitoring might be considered a poor use of podiatric resources for low‐risk events, the benefit of potentially catching early skin degenerative changes is probably outweighed by any extra workload; furthermore, viewing a scan and making a clinical decision is a relatively quick process.

Podiatrists considered visual images useful much more often than thermal data in when reviewing reports (90% vs. 12%, respectively). Potential reasons for this are: firstly, the relatively low impact of the thermal data probably reflects the relatively infrequent nature of large plantar delta changes (> 2.2°C) occurring in the natural history of the diabetic foot. Indeed, only 8% of our reports were primarily created due to ‘hotspots’. In other pivotal studies of at‐home daily foot temperature monitoring, large delta changes were also infrequent, with only 3–4.5 hotspots/person‐year detected, and one‐third of all high‐risk patients not having a single hotspot during an 18‐month follow‐up [16, 17]. Furthermore, although temperature‐based analyses may be useful as a markers for future skin breakdown, their use is hampered by high false positive rates (57%), that is, low specificity for DFU prediction [23]. Nevertheless, temperature monitoring remains an important aspect of preventative footcare, and our data have shown good feasibility for acquiring daily temperature measurements in high‐risk patients. Secondly, our data illustrate the relative importance of visual images for identifying potential precursors to skin breakdown, such as callus build‐up or presence of foreign bodies occurring during daily life, compared with rarer delta changes. Thirdly, the early clinic referrals made here following visual images may have pre‐empted any active inflammation. If left alone, these issues may have developed into a heat signal.

The remote Monitoring System correctly interpreted plantar skin issues compared to the gold standard in‐clinic patient examinations, to a good level, and with high sensitivity (80%) and specificity (100%), providing a useful validation of the system to detect precursors for DFU in future studies. There is current interest in the potential use of digital photographic images to assist in clinic‐based diabetic foot care, via telemedicine, assisting close patient monitoring and two‐way communication with healthcare providers to improve outcomes. The implication is that remote monitoring using digital images may not only improve DFU healing times but may also prevent DFU occurrence, initiated by the early flagging of pre‐ulcerative lesions [22, 31].

There were three cases of direct podiatry intervention here, following report review, which we consider may have pre‐empted and prevented incident skin breakdown/DFU: (a) visual evidence of a ‘foreign body’, prompting an urgent clinic visit at which a small stone was found lodged into the apex of the hallux and removed without skin break; (b) visual evidence of a coin lodged to plantar foot surface, prompting same day telephone contact and footcare advice; (c) large thermal delta rises, prompting a clinic visit at which severe callus was debrided from the site of the hotspot.

During the study, 26% of participants developed new skin lesions over 12 weeks. Although a higher rate than expected for this cohort [2], the majority of these DFUs were superficial (Grade 1A). The monitoring system visual scans correctly flagged and identified 100% of these incident DFUs and crucially the system was the first point of detection for over half of them, otherwise unknown to standard podiatry services, showing the potential usefulness of the device for very earliest DFU detection. Although only a relatively small percentage of scans (5%) caused a report to be generated, the system's time and resource requirements for daily monitoring may be justified as all incident DFU were quickly identified by this protocol.

There were some limitations to the study: (1) We have not provided here estimates and costings of potential DFU reduction in a larger RCT as our focus was the feasibility of patient device use and monitoring system procedures; however, our finding that the scans were the first point of detection for 50% of the new DFUs should enable new costing estimates for increases in ulcer‐free days in future RCTs. In a future system, both monitoring and patient outreach will be carried out by Bluedrop Medical. Ultimately, the company intends to automate and optimise aspects of this service to be able to deliver the service (including use of the device), at a cost neutral or cost saving price point to the health system. (2) The scan visual images included the presence of ‘dots’, created by the eTLC sensors, potentially obscuring sites of DFU/skin issues. Podiatrists recorded, however, that the dots did not impair the interpretation of the scan visual images in 93% of scan reports. (3) Two patients were withdrawn early due to damage to the device caused by unknown factors. (4) On one occasion, scan images were not feet of the consented participant but those of a family relative living in the same house. Future software changes, however, will enable observer visual recognition of baseline foot images on all scans to prevent the effect of this. (5) There may be potential risks/complications from using the scanner including patient slips or falls when stepping on or off the scanner, especially because of lower‐than‐expected balance issues in this cohort; however, none occurred in study. (6) Trips away from home may have resulted in significant periods without remote foot monitoring for some patients. Anecdotally, several of our participants reported that they had taken their device with them whilst on holiday and had continued to take daily scans. This suggests that the device has reasonably good portability and patients could be encouraged to do this to maximise their remote foot monitoring. (7) The time interval between the identification of thermovisual abnormalities and clinical instruction to the patient could be improved. This limitation was a result of our protocol of centralized review followed by podiatrist‐led action, which could take up to 1–2 days. In response to this, the BMS has since been refined to include a structured clinical escalation protocol that enables same‐day direct contact with the patient in specific scenarios. When scan abnormalities (such as a sustained temperature delta ≥ 2.2°C or visual signs suggestive of pre‐ulcerative changes) are identified, trained personnel within the monitoring service now initiate immediate patient outreach and provide first‐line guidance, including instructions to reduce weight‐bearing activity or initiate basic foot protection measures. This action is taken prior to formal podiatric review when appropriate, based on predefined clinical criteria informed by the pilot study experience. The goal of this update is to shorten the delay to intervention, enabling earlier clinical action and patient self‐care in accordance with DFU prevention guidelines, without compromising safety.

In conclusion, we have demonstrated a high level of patient usability and clinical utility of a remote visual and thermal foot scanning and monitoring digital system, with human touch patient engagement and provision of healthcare provider support. The system correctly flagged and identified all new DFUs occurring during the study and was the first point of detection for over half of these ulcers, which were otherwise unknown to standard podiatry services. Remotely assessed scan visual images could determine plantar areas of concern, pre‐ulcerative lesions or DFU in a manner comparable to that of the in‐clinic podiatric foot assessments, showing good sensitivity (80%) and specificity (100%). The unique multi‐modal (thermal and visual) sensing feature of the system allows detection of skin problems across the aetiological window of DFU development, from the tipping point of tissue inflammation and callus build‐up, which should enable rapid intervention, to the earliest detection of full thickness skin break, which should enable rapid DFU treatment. The findings from this study will inform the design of a larger randomised controlled trial to develop quantitative evidence regarding DFU prevention using a remote monitoring system alongside standard podiatric services.

## Author Contributions

C.A.A. and A.J.M.B. conceived and designed the study, C.A.A., C.M. and A.J.M.B. supervised the study, K.J.F., D.E.S., E.K. and S.F. recruited participants, K.J.F., D.E.S., E.K., S.F., R.S. and C.A.A. performed study investigations, C.A.A. formally analysed the data and wrote the original manuscript draft. C.A.A. and A.J.M.B. reviewed and edited the manuscript. All authors have read and approved the final version of the manuscript.

## Conflicts of Interest

R.S. received partial funding from Bluedrop Medical, the company acting as sponsor for the study and providing study devices and financial support to the clinical sites for study operations. There are no other potential conflicts of interest relevant to this article.

## Peer Review

The peer review history for this article is available at https://www.webofscience.com/api/gateway/wos/peer-review/10.1002/dmrr.70096.

## Data Availability

The data that support the findings of this study are available from the corresponding author upon reasonable request.
